# Cardiac pacing challenge in Sub-Saharan Africa environnement: experience of the Cardiology Department of Teaching Hospital Aristide Le Dantec in Dakar

**DOI:** 10.1186/s12872-019-1176-2

**Published:** 2019-08-14

**Authors:** Adama Kane, Simon Antoine Sarr, Juliette Valerie Danièle Ndobo, Alioune Tabane, Kana Babaka, Fatou Aw, Malick Bodian, Serigne Mor Beye, Momar Dioum, Aliou Alassane Ngaidé, Mouhamadou Bamba Ndiaye, Alassane Mbaye, Maboury Diao, Abdoul Kane, Serigne Abdou BA

**Affiliations:** 1 Cardiology departement of teaching Hospital Aristide Le Dantec, Dakar, Senegal; 2 Gaston Berger University, Saint-Louis, Senegal; 3 Cardiology departement of Grand Yoff Hospital, Dakar, Senegal

**Keywords:** Cardiac pacing, Pacemaker, Senegal

## Abstract

**Background:**

Cardiac pacing is a growing activity in Sub-Saharan Africa. There is little data on the characteristics of this interventional treatment in our regions. The goal was to evaluate the results of cardiac pacing in a referral service in sub-Saharan Africa.

**Methods:**

We carried out a twelve-year retrospective study (from January 1st, 2004 to December 31st, 2015) in the Cardiology Department of Aristide Le Dantec University Hospital. This work included all patients who received definitive cardiac pacing during the study period and followed up in the service.

**Results:**

In total we included 606 patients. There was a growing trend in activity with a peak in 2015 (17%). The average age was 70.6 ± 12.03 years. Some patients (15.4%) came from the subregion. The patients were mostly of medium socio-economic level (53%); 14% were of low socio-economic level. Patients were symptomatic in 85% of cases (37.4% syncope). The indications were dominated by complete atrioventricular block (81.5%); sinus dysfunction accounted for 1.9% of them. A temporary pacemaker was used in 60% of cases for an average duration of 5.1 ± 6.3 days. Antibiotics, local anesthesia and analgesics were used in all cases. Implanted pacemakers were single chamber in 56% of cases and double chamber in 44% of cases. In 39 patients (6.4%), the pacemaker was a « re-used » one. The atrial leads were most often placed in a lateral position (94.5%). The ventricular ones were predominantly tined (95.7%) and more often located at the apical level. Complications were noted in 24 patients (3.9%), dominated by devices externalizations and infections, which together accounted for 2.7% of cases. The number of people in the cathlab was significantly higher and the duration of the temporary pacemaker was longer for patients who had a complication. There was no significant difference depending on the type of pacemaker used (new or reused). Seven (7) in hospital death cases were reported.

**Conclusion:**

Cardiac pacing is a growing activity in Dakar.

## Background

Pacing is a lifesaving treatment during cardiac conduction disorders. Patients with these disorders are exposed to heart failure but also to sudden death due to too long asystole or ventricular arrhythmia resulting from significant bradycardia [1]. There is a growth activity in our sub-Saharan African countries. However, the progression is impeded, on one hand, by the high cost of cardiac implantable electronic devices CIED [2] and the lack of medical insurance for patients, and on the other hand, by the limited or nonexistent human resources depending on the country [3]. In addition, pacing exposes to many complications, sometimes serious as infection. Few data are available for cardiac pacing in Sub-Saharan Africa.

The objective of this study was to evaluate cardiac pacing in a reference service in West Africa. The specific objectives were: to describe the indications for cardiac pacing, to evaluate its practical modalities and to identify complications and predisposing factors.

## Methods

This work was carried out in Senegal, in the Cardiology Department of the Aristide Le Dantec University Hospital Center in Dakar. It is a reference service for cardiac pacing. It has 48 beds, 4 of which are in the intensive care unit. It is equipped with two cardiac catheterization rooms:

- An angiography room with a SIEMENS image intensifier with motorized table.
- A General Electric Healthcare Innova 3100-QIPlus room acquired in 2013.

These rooms are equipped with 3 temporary pacemakers, two of which are Medtronic brand and one Biotronik, and different programmers (Medtronic, Merlin of Saint Jude Medical, Ela Sorin Group, and Boston). The second is equipped with a Lap System pros electrophysiology.array with a multiparameter scope that allows electrophysiological exploration and.radiofrequency ablations.

On the staff side, there is a Professor specialized in rhythmology and cardiac pacing, and five cardiologists able to implant pacemakers, who have been trained on the spot.

This is a retrospective study over twelve (12) years: January 1st, 2004 to December 31st, 2015.

The inclusion criteria were: all patients who received definitive cardiac pacing during the study period in the cardiology department of ARISTIDE LE DANTEC.

All patients who received triple chamber pacing and those who had an implantable cardioverter defibrillator were excluded. Patients whose records were unusable, as well as those who were implanted in the service but followed in other structures were excluded to.

Data sources were patient records, electronic operative records, pacemaker room records and post-implantation dressing care, collection tools (it was a counting sheet).

Studied parameters were:

- Socio-demographic data: age, gender, socio-economic level
- Clinical data: antecedents and grounds, symptoms
- Electrocardiographic Indications: Type and Degree of Conduction Disorder
- Characteristics of the implantation: context, type of act, implantation site, approach, position of the leads, incidents, accidents and operational difficulties
- Pacemaker and leads characteristics: type, new or reused, leads characteristics
- Evolution: complications (nature, delays, and management) and the favoring factors.

The data were collected on the survey form attached. The input and analysis were made respectively on the software Sphinx version 5.1.0.5, on Epi info version 3.5 and EXCEL 97–2003. The bivariate analysis made it possible to determine the distribution of each variable. We compared the group of patients who had complications to those who did not have them in order to determine the contributing factors. Quantitative data were expressed as an average. The chisquare statistical test (*p* value) was used to compare the variation of the different parameters as a function of time and to perform crosses. It was considered significant for a value of *p* < 0.05.

The need for ethics approval for this study is deemed unnecessary according to actual national.

regulations in Senegal.

## Results

**Fig. 1:**
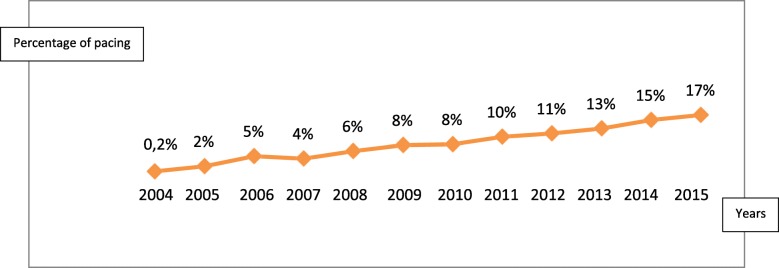
Evolution of cardiac pacing

**Fig. 2:**
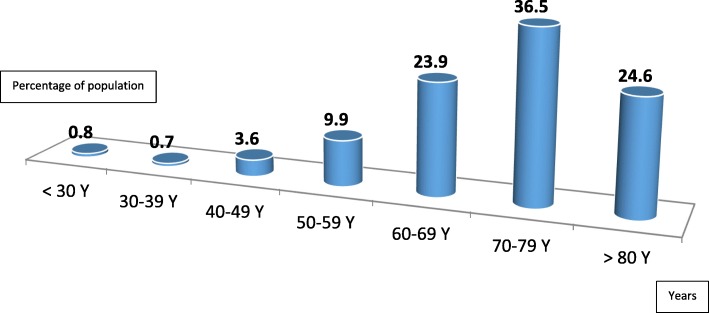
Population distribution by age

In total we included 606 patients implanted in this study. There was a change in activity with a peak in 2015 (17%) as illustrated in the Fig. 1. The average age of the patients was 70.6 ± 12.03 years with extremes of 17 and 98 years. The age group 70–79 was the most represented (36.5%) as shown in Fig. 2. Women were majority and accounted for 52.3% with a sex ratio = 0.52. The majority of patients (513 patients or 84.6%) were from Senegal, most often from Dakar (411 patients). Some of them (15.4%) came from the sub-region (Gambia, Mali, Mauritania, Guinea Conakry, Guinea Bissau, Togo, Cameroon, Sierra Leone). The patients were mostly of medium socio-economic level (53%); 33 and 14% were of good and low socio-economic level respectively. Stimulated patients were hypertensive in 79% of cases; 24% of them were known to be diabetic. In addition, 8% had a history of stroke and 11.8% had left systolic dysfunction. Most of them (85%) had symptoms such as syncope (37.4%), vertigo (57.5%), lipothymia (4.7%) and exercise dyspnea (21%). Indications were dominated by complete atrioventricular block (AV block) (81.5%). Sinus node dysfunction (SND) accounted for 1.9% of indications. Table 1 summarizes the various indications.

**Table 1:** Frequency of indications

Indications	Number (*n* = 606)	Poeurcentage Percentage(%)
Third degree AV block	493	81,5
High grade AV block	86	14,2
Sinus node dysfunction	12	1,9
Trifascicular block	12	1,9
Second degree AV block	3	0,5

*AV* atrioventricular

Cardiac pacing was urgently performed in 139 patients (22.7%). It was mainly a primoimplantation (87.7%). The cathlab team members were on average 3 ± 0.6 with extremes of 2 and 6. A temporary pacemaker (before definitive pacing) was used in 60% of cases for a duration on average of 5.1 ± 6.3 days with limits of 1 and 30 days. The implantation site was essentially pre-pectoral left (98.7%). It was pre-pectoral right in 1.3% of cases. In 52.6% of patients, the subclavian approach was used; the cephalic route was used in 47.4% of our patients over the entire study period. However, we noted an increase in the use of the cephalic approach, which has been used more than sub-clavian one since 2011. Antibiotics (third generation cephalosporins), local anesthesia and analgesics were administered to all patients.

Implanted pacemakers were single chamber in 56% of cases and double chamber in 44% of cases. These were new CIED in 93.6% of cases. In 39 patients (6.4%), there was reused pacemakers. Atrial leads were bipolar in 99.5% of the cases and mostly screwing leads (98.5%). These were most often placed in a lateral position (94.5%); the others (5.5%) were located at the auricle. Ventricular leads were bipolar in 96.7% of cases and predominantly tined (95.7%). They were most often placed at apex (99%); the rest was in the septal position. For the per-procedural collections, the average impedance and threshold of the atrial lead were 661.4 ± 165.3 Ohm and 0.40 ± 0.32 V, the pulse duration was 0,4 milliseconds. For the ventricular lead, it was respectively 891.6 ± 227.1 Ohm and 0.32 ± 0.24 V, the pulse duration was 0,4 milliseconds. Per-procedural incidents and accidents were reported in 8.3% of patients. Syncopal status and complications of local anesthesia were most common (Table 2). Complications were noted in 24 patients (3.9%), dominated by devices externalizations and infections, which together accounted for 2.7% of cases. The average time of onset was 175 and 200 days, respectively (Table 3).

**Table 2:** Frequency of per-procedural incidents and accidents, and implantation difficulties

Type	Frequency (*n* = 43) (n)
Incidents	39
Syncope	16
Agitation due to local anesthesia	8
Bleeding	6
Vagal type reaction	4
Atrial lead dislodgment after surgical	2
Hypoglycemia	1
Substain rythm disturbance	2
Accidents	4
Cardiorespiratory arrest	2
Lead rupture (battery replacement)	1
Cardiac tamponade due to right atrium perforation	1
Major implantation difficulties	
Manufacturing connector fault	2
Problem of leads disconnection (battery replacement)	2
Problem of atrial lead placement	1
Failure of temporary pacemaker placement	1

**Table 3:** Frequency of complications

	Frequency (*n* = 33)	Percentage	Delay (days)
Complications (n)		(5,4%) (%)	
Infections	8	1,3	175 ± 259,7
Pocket hematoma (requiring further surgery)	4	0,6	2 ± 0,8
Upper extremity deep venous thrombosis	3	0,4	16 ± 2,8
Pacemaker externalizations	9	1,4	200 ± 148,5
Leads dislodgment	4	0,6	90 ± 45,8
Pneumothorax	4	0,6	1 ± 0,3
Lead fracture	1	0,16	1095

**Fig. 3:**
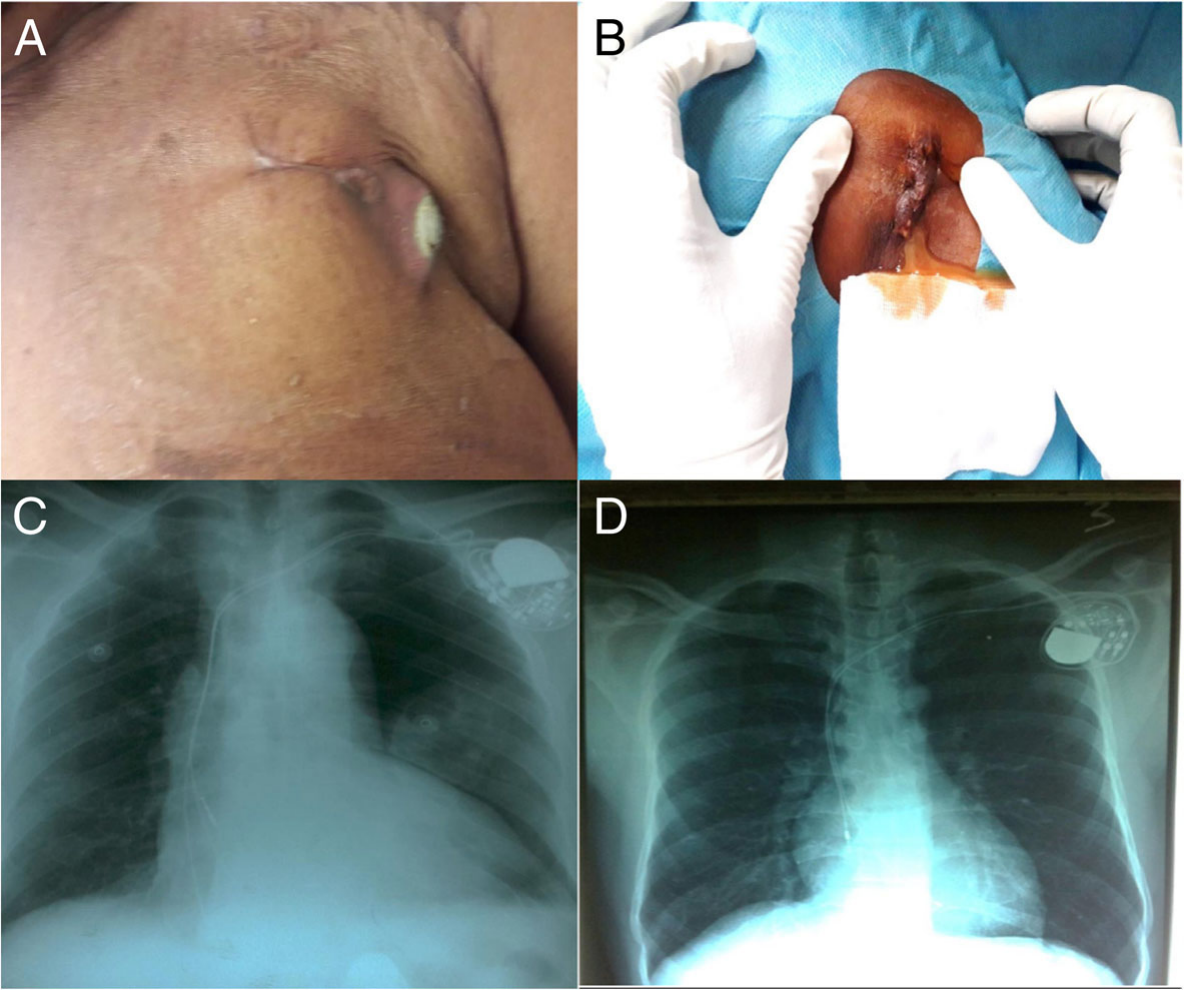
Picture compilation of complications (infection, externalization and leads dislodgement). A Externalization of pacemaker. B Infection of pocket. C Right ventricle lead’s dislodgement. D Right atrial lead’s dislodgement

In one case, it was a complete lead fracture associated with pectoral stimulation. The Fig. 3 illustrates some of the noted complications.

Patients who had complications were significantly older than those who did not have any: 76.6 ± 9.7 years vs 70.4 ± 12.0 years (*p* = 0.03). The systolic dysfunction of the left ventricle was significantly related to the occurrence of complications. The same was true for the occurrence of per-procedural incident or accident (*p* = 0.01). The number of persons in the cathlab was significantly greater and the duration of the temporary pacemaker was longer for patients who had a complication versus those who did not (p respectively 0.02 and 0.04). There was no significant difference according to the use of new or reused pacemakers (*p* = 0.57).

In post-pacing, complications were significantly related to the onset of fever (*p* = 0.03), pacemaker’s box pain (*p* = 0.03) and lipothymia (0.014). Seven (7) cases of death during hospitalization were recorded (1.1%). These patients had a lot of comorbidities, most often a stroke. The death was attributable to the procedure in one case. This was an 80-year-old patient who had an irreducible ventricular tachycardia followed by cardiac arrest as soon as the ventricular lead was introduced.

## Discussion

Our work is a report of activity concerning a well-developed interventional technique in the Western countries, but emerging in some West African countries. However, it is the only effective treatment of severe cardiac conduction disorders showing, therefore, the need for the development of this activity. There has been an increasing growth in the number of implantations during the last 12 years, with a peak in 2015. This increase in activity is linked to several parameters:

- Human resources: the staff includes, for several years, a professor graduated in rhythmology and pacing which has strengthened its status as a national or even sub-regional reference service.

- The development of electrophysiological exploration played an important part.

- Specialized training and the creation of regional cardiology departments have led to a greater number of diagnosed cases.

These figures probably place our service among those with greater activity in sub-Saharan Africa. Falase reported 51 pacemaker implantations in 5 years in a university hospital in Nigeria [2]. In 2013, a total number of 16,271 devices were implanted in 21 countries in Africa, representing an implantation rate of 18 devices per million population; whereas in 2014, a total number of 11,600 devices were implanted in 17 countries, representing an implantation rate of 19 devices per million population. The number of pacing centers per million population was < 1.0 in 2013 and remained unchanged in 2014 [3]. We are still far from the very important activity of some Western services [4]. This difference can be explained by the high cost of equipment in a low-income population and mostly without medical insurance.

In addition, qualified human resources in this area in sub-Saharan Africa is a real problem: at best the number of people able to implant a pacemaker is very limited, at worst these resources are nonexistent. The implantation is then done during missions carried out by foreign specialists or the patients are sent abroad to be implanted there [3].

We noted a significant increase in activity that seems to be related to the presence of a specialist. It therefore seems imperative to create training niches for a more important activity in our countries, for the benefit of the patients.

Beyond this, the use of sterilized pacemakers commonly known as “re-use” seems to be a sure.

way and would solve the problem of the accessibility of CIED [5]. Moreover, 6.4% of.

patients have benefited from this type of pacemaker in our series.

The average age of the patients was 70.6 ± 12.03 years. This result is similar to that reported in Nigeria [2] but in developed countries, this age seems more. This is undoubtedly the fact of a higher life expectancy.

Our work shows that symptomatic complete atrioventricular block is the most common indication. Syncope was a common symptom (37.5%). The frequency of complete atrioventricular block is reported in most series in sub-Saharan Africa [2]. In developed countries, the finding is different: disorders of atrioventricular conduction of lesser degree and sinus dysfunction have a greater magnitude. In the Italian Association of Arrhythmia and Pacemaker Implantation Registry, atrioventricular block accounted for only 23.8% of indications, SND and bradycardia associated with atrial fibrillation respectively in 22.9 and 15.1% [6]. Sinus dysfunction was only 1.9% of indications in our series.

However, despite the urgency of the implantation in a large part of the indications, the installation of the pacemaker was immediately possible only in 22.7% of the cases because of the financial impossibility of the patient to buy the cardiac implantable electronic device (CIED) on admission. In addition, the lack of qualified human resources partly explains the predominance of serious conductive disorders as indications [3]. In fact, SND is often under-diagnosed in our country because of minimization of symptoms and a lack of knowledge about ECG signs of SND.

In terms of the technique of pacemaker placement, there has been a clear evolution with the increasing use of the cephalic vein at the expense of subclavian puncture which exposes more complications. This work shows a significant use of the temporary pacemaker (60%) and, moreover, for a long duration (5 days on average). This is explained on the one hand by indications consisting mainly of symptomatic complete atrioventricular block of which 37.4% were syncope. On the other hand, the temporary pacemaker is often placed in front of the urgency of severe symptomatic bradycardia, positive chronotropic drugs are not available and patients can not afford the pacemaker. Whereas the temporary pacemaker exposes to several complications: infections, thromboembolic risk, secondary displacement, battery usury [1]. Implanted pacemakers were single chamber in 56% of cases and double chamber in 44% of cases. Falase reported the same proportions. The proportion of double-chamber cells is much higher in developed countries as in Italy: 64% versus 26.9% for mono-chambers (the rest consists of automatic implantable defibrillators and resynchronization) [2]. This situation can be explained by the higher cost of dual chamber pacemakers in our regions (on average 200 to 500 US dollars more expensive) [2, 3]. Although dual chamber pacing, which is more physiological, gives more benefit (lower risk of atrial fibrillation and stroke). It should be noted that there is no significant difference in mortality.

In addition, dual chamber pacing involves a longer procedure and exposes to more complications [6, 7]. In FOLLOWPACE study, within 2 months, 12.4% patients developed PM complications mostly lead-related complications [8].

These complications are the obsession in cardiac pacing due to their severity and the difficulty of their management. Infections and externalizations were the main complications of our series (2.7%). These are one of the most worrying post-operative complications which can lead to endocarditis [9, 10]. This is a real problem in our regions. Patients hardly acquire the CIED, and therefore conservative treatment is often the only solution. In a review of the literature including 60 works, Polyzos reported a frequency of CIED infection of 1–1.3%. Risk factors were diverse [11]:

- Diabetes, end-stage renal disease, chronic obstructive pulmonary disease, glucocorticoid therapy, renal failure, cancer, history of prosthetic infections, heart failure and fever, anticoagulation, skin conditions.
- Regarding the procedure: hematoma, re-intervention for probe displacement, replacement of the CIED, lack of antibioprophylaxis, temporary pacemaker, lack of experience of the operator.
- Regarding the specificities related to the CIED: the abdominal pocket, the placement of epicardial probes, the placement of two or more probes and the double chamber pacemaker.

## Conclusion

Pacing is a growing activity in our practice. Its development is however impeded, in large part, by the high cost of cardiac implantable electronic devices. Symptomatic complete atrioventricular block is the most important indication. The complications are dominated by infections and externalizations.

## Data Availability

The datasets used and/or analysed during the current study are available from the corresponding author on reasonable request. The data we received were de-identified.

## Abbreviations

AV block: Atrioventricular block
